# La salud mental en Colombia, ¿es un derecho o un privilegio?

**DOI:** 10.7705/biomedica.7429

**Published:** 2026-06-01

**Authors:** Martha Escobar, Álvaro Arenas, Camilo Perdomo-Luna, Darío Londoño, Juan Carlos Molano, María Nieto, Elizabeth Borrero, Esteban Vanegas

**Affiliations:** 1 Dirección de Salud Poblacional, Fundación Santa Fe de Bogotá, Bogotá, D. C., Colombia Dirección de Salud Poblacional Fundación Santa Fe de Bogotá Bogotá, D. C. Colombia; 2 Departamento de Salud Mental, Fundación Santa Fe de Bogotá, Bogotá, D. C. , Colombia Departamento de Salud Mental Fundación Santa Fe de Bogotá Bogotá, D. C. Colombia; 3 Facultad de Medicina, Universidad de los Andes, Bogotá, D. C., Colombia Universidad de los Andes Facultad de Medicina Universidad de los Andes Bogotá, D. C. Colombia

**Keywords:** salud mental, gastos en salud, promoción de la salud, Colombia, Mental health, health expenditures, health promotion, Colombia

## Abstract

Este artículo ofrece una visión de la situación de la salud mental en Colombia, destacando su creciente relevancia en la esfera pública y los desafíos específicos que enfrenta el país al respecto. Asimismo, analiza el impacto del conflicto armado y la pandemia de la COVID-19 en la salud mental de la población colombiana, subrayando cómo estas situaciones han exacerbado los problemas preexistentes. Además, resalta la existencia de barreras significativas para acceder a los servicios especializados, como son el estigma, la falta de personal capacitado y los altos costos asociados con el tratamiento de los trastornos mentales.

Por lo anterior, se plantea la necesidad de abordar las desigualdades en la atención de la salud mental, tanto a nivel nacional como internacional. Finalmente, se proponen recomendaciones para mejorar la prevención, la atención comunitaria y el acceso a los servicios de salud mental en Colombia.

## Abstract (en)

This article offers a vision of the mental health situation in Colombia, highlighting its growing relevance in the public sphere and the specific challenges facing the country. It analyzes the impact of the armed conflict and the COVID-19 pandemic on the mental health of the Colombian population, focusing on how these situations have exacerbated pre-existing problems. Additionally, the existence of significant barriers to accessing specialized services is highlighted, such as stigma, lack of trained personnel, and high costs associated with the treatment of mental disorders.

Therefore, the need to address inequalities in mental health care is raised, both nationally and internationally. Finally, recommendations are proposed to improve prevention, community care and access to mental health services in Colombia.

La salud mental, según la Organización Mundial de la Salud (OMS), es un estado de bienestar en el que las personas pueden relacionarse, desempeñarse en la vida cotidiana, adaptarse a los cambios y prosperar frente a las dificultades, lo que contribuye a una mayor productividad y mejor calidad de vida ^1^^,^^2^. Por el contrario, la presencia de alteraciones en las esferas cognitivas, emocionales o conductuales que afectan la funcionalidad de la persona y la capacidad de avanzar en el ciclo vital, puede llegar a constituir una enfermedad mental.

Estos fenómenos deben entenderse no solo desde la clínica individual, sino dentro de un marco teórico que articule los múltiples factores determinantes sociales de la salud y el funcionamiento de los sistemas de salud mental, como lo proponen los WHO-AIMS (*World Health Organization Assessment Instrument for Mental Health Systems*) en la evaluación de políticas, recursos y servicios. Este enfoque permite analizar la salud mental como un proceso complejo, influido tanto por condiciones individuales como por factores estructurales y contextuales.

El aumento de las enfermedades mentales en Colombia resulta alarmante. En los últimos cinco años, el número de consultas por temas de salud mental se incrementó en el 34,5 % ^3^. Según la Encuesta Nacional de Salud Mental y la Encuesta de Salud Mental de Bogotá 2023, cerca del 80 % de la población reporta síntomas de depresión y el 53 %, síntomas de ansiedad, mientras que el 44,7 % de los niños presenta indicios de algún problema mental ^4^. Estas cifras demuestran una afectación transversal en distintas etapas de la vida. En particular, la conducta suicida en Colombia continúa en aumento y afecta de manera predominante a los jóvenes de los 15 a los 29 años, con una tasa nacional de 6 por cada 100 000 habitantes ^5^.

La OMS predice que, para el año 2030, la enfermedad mental será la principal causa de enfermedad en el mundo ^6^ y advierte que, sin la implementación de estrategias urgentes y radicales, esta proyección se cumplirá en menos de lo previsto. Estos datos reflejan una crisis de salud mental global que se encuentra en proceso de agudización y que se acompaña de barreras estructurales como el desconocimiento, el estigma y el autoestigma, la falta de personal especializado, la escasez de servicios y los altos costos asociados con el manejo de los trastornos mentales. En este contexto, Colombia no es la excepción: aunque las políticas nacionales, como la Ley 1616 del 2013, establecen que la salud mental debe abordarse de manera integral, garantizando la atención de todas las necesidades de los pacientes ^7^, la realidad muestra un panorama distinto. La atención psiquiátrica en el país presenta falencias entre las que se destacan la insuficiente infraestructura y el limitado número de profesionales capacitados ^8^.

El tratamiento inadecuado o la falta de tratamiento de las condiciones mentales pueden aumentar la probabilidad de que las personas consuman diversas sustancias, tengan dietas nutricionalmente inadecuadas y no cumplan con los niveles recomendados de ejercicio ^9^^).^ Esto, a su vez, aumenta la obesidad y la dislipidemia, la resistencia a la insulina, la diabetes y la hipertensión, que son importantes factores de riesgo de enfermedad cardiovascular, la principal causa de muerte en el mundo ^10^^,^^11^.

Por otro lado, las enfermedades crónicas como el cáncer, las enfermedades cardíacas y la diabetes, pueden desencadenar condiciones psiquiátricas y llevan a la depresión y la ansiedad ^11^.

Se estima que, en Colombia, el 21,4 % de la población entre los 18 y los 44 años presenta comorbilidad médico-psiquiátrica, es decir, la coexistencia de al menos un trastorno mental (como depresión o ansiedad) junto con una o más enfermedades crónicas no transmisibles, tales como hipertensión, diabetes o enfermedad cardiovascular ^12^. Esta prevalencia resulta de gran importancia para el panorama nacional de salud mental y, al no considerar a los adultos mayores, quienes suelen acumular más condiciones médicas y psiquiátricas, es probable que el porcentaje real sea aún mayor.

El panorama de la salud mental nacional tiene dos agravantes importantes: el conflicto armado del país y la pandemia por la COVID-19 (c*oronavirus disease-*2019). En el primero, se hace referencia al conflicto armado interno que se ha prolongado por más de cinco décadas y que, según el Registro Único de Víctimas, ha dejado más de 9 millones de personas afectadas, incluyendo aproximadamente 450 000 muertes, 51 000 secuestros y cerca de 8,5 millones de desplazamientos forzados entre los años 80 y el 2018 ^13^. Este impacto ha generado profundas secuelas emocionales y sociales en las comunidades afectadas.

La literatura académica internacional señala que las prevalencias de depresión, ansiedad y trastorno de estrés postraumático son de dos a tres veces más altas en las personas expuestas a los conflictos armados, siendo las mujeres y los niños los más vulnerables ^14^. En los estudios nacionales, también se ha identificado un aumento significativo de la prevalencia de síntomas y trastornos mentales en víctimas del conflicto, particularmente en desplazados internos, quienes enfrentan, además, barreras de acceso a los servicios de salud y condiciones de vulnerabilidad socioeconómica ^15^.

El segundo agravante ha sido la pandemia de la COVID-19, en la cual, por más de dos años, los gobiernos del mundo concentraron su atención y destinaron sus ya limitados recursos en salud a contener el virus, dejando en un segundo plano otra pandemia en crecimiento: la de las enfermedades mentales, que ha cobrado millones de vidas y ha generado discapacidad en miles de personas ^16^^,^^17^. El impacto fue especialmente grave en niños y adolescentes, quienes sufrieron las consecuencias de la suspensión de las clases presenciales, las alteraciones de su rutina diaria, la pérdida de interacciones sociales, la mayor exposición a la violencia intrafamiliar y la escasa priorización de la atención en salud mental, entre otros factores ^17^.

Ante estos dos agravantes, Colombia cuenta con la Política Nacional de Salud Mental. Este instrumento busca garantizar condiciones de equilibrio y bienestar humano para un desarrollo integral, desde un enfoque de capacidades y derechos humanos, reconociendo la salud como un derecho fundamental. Sin embargo, el acceso a los servicios de salud mental, incluyendo los orientados a la prevención y a la promoción, continúa siendo un privilegio de pocos.

Por lo anterior, el incremento de los trastornos mentales requiere de acciones que puedan contrarrestar las diferentes brechas que afectan la atención en salud mental, como las de información, políticas y de legislación, recursos (presupuesto, personal entrenado y medicamentos, entre otros) y servicios. Estas acciones deben incluir la implementación de estrategias por fuera de los consultorios, es decir, en el contexto comunitario, en el cual más se necesita y donde las intervenciones son más costo-efectivas ^2^.

Un ejemplo de ello lo constituyen los programas de atención psicosocial desarrollados en las comunidades afectadas por el conflicto armado en Colombia, como el Programa de Atención Psicosocial y Salud Integral a Víctimas (PAPSIVI), los cuales han demostrado efectos positivos en la reducción de los síntomas de depresión y ansiedad, además de facilitar la reconstrucción del tejido social en entornos de gran vulnerabilidad ^18^^).^ De manera similar, las experiencias internacionales como el modelo británico *Improving Access to Psychological Therapies*, han demostrado que el brindar servicios psicológicos comunitarios logra mejorar los resultados en salud mental a un costo significativamente menor que la atención hospitalaria especializada ^19^.

Además, el enfoque preventivo mediante la implementación de los programas de atención comunitaria podría evitar algunos de los síntomas de la depresión -como poca productividad, apatía o falta de energía- que cuestan, a nivel mundial, cerca de USD$ 1 000 000 000 000 anuales por concepto de pérdida de productividad ^20^.

A continuación, se describen algunos conceptos y estadísticas fundamentales para ahondar y explicar mejor la magnitud de lo que genera el aumento de la enfermedad mental.

## Carga de salud mental

Según la OMS, la carga de una enfermedad se define como «el impacto de un problema de salud en un área específica que se mide por la mortalidad y la morbilidad» ^21^. Esta se puede evaluar en términos de años de vida ajustados por discapacidad, años perdidos por discapacidad, años perdidos por muerte prematura y años saludables perdidos por discapacidad o muerte prematura ^21^. Por consiguiente, la discapacidad se mide mediante un puntaje de gravedad entre 0 y 1, donde 0 representa perfecta salud y 1 representa la muerte.

La enfermedad mental implica también una carga significativa para la familia y el entorno del paciente, lo que se traduce en un costo social amplio. Según los informes de la OMS, los trastornos mentales no tratados representan el 13 % del total de la carga de la morbilidad mundial ^22^. La depresión unipolar, por ejemplo, se encuentra entre las principales causas de morbilidad: ocupa el tercer lugar y representa el 4,3 % de la carga global ^21^. En términos prácticos, esto refleja que la depresión es una de las condiciones más incapacitantes y una de las causas por las que más años saludables pierde la población ^23^.

Finalmente, es importante resaltar que la salud mental no depende únicamente de factores biológicos o clínicos, ni puede entenderse como un estado binario de salud o enfermedad. En vez de eso, se trata de un continuo que influye en múltiples dimensiones de la vida de una persona y, por tanto, requiere abordajes integrales. De allí surge la necesidad de un enfoque multisectorial y colaborativo que involucre al sector laboral, educativo, de vivienda, comunitario, digital e incluso legal, entre otros ^24^.

## Gasto en salud

El gasto en salud es la suma de los costos incurridos en la totalidad de los servicios de atención de salud, cuyos fondos se manejan por parte del gobierno o del sector privado. Los servicios incluyen, entre otros, los bienes médicos dispensados a los pacientes ambulatorios, los servicios de prevención y de salud pública, la administración de la salud, los seguros médicos, las actividades de planificación familiar, las actividades de nutrición y la ayuda de emergencia asignada para la salud. Este indicador se expresa como un porcentaje del producto interno bruto (PIB), como la proporción del gasto total en salud y, además, puede convertirse a una unidad común, como dólares per cápita ^25^.

En el panorama internacional, según el atlas de salud mental de la OMS del 2024, en promedio solo el 2,1 % del gasto total en salud se destina a la atención de la salud mental. Esta información financiera específica, se recolecta de 75 de los 144 países miembros entre julio del 2024 y marzo del 2025. Se encuentra que en los países de ingresos altos este valor puede alcanzar el 4,83 %, mientras que en los de ingresos medios bajos el presupuesto promedio asignado fue del 1 % ^26^.

Según la OMS, la brecha de acceso entre las personas con alto poder adquisitivo y aquellas con uno bajo agrava la situación: quienes tienen menos recursos presentan mayor riesgo de padecer esquizofrenia, depresión, ansiedad y uso de sustancias psicoactivas ^2^. Estos problemas no se limitan a los países de bajos ingresos; incluso donde hay recursos disponibles, la salud mental no goza de paridad con la salud física en la asignación presupuestaria ^2^. Asimismo, se ha descrito una relación estrecha entre la enfermedad mental y la pobreza, que opera como un círculo vicioso: la enfermedad mental genera más pobreza y la pobreza incrementa la probabilidad de enfermar ^2^.

En este contexto, los gastos de salud asociados con las enfermedades físicas, frente a las enfermedades psiquiátricas, revelan diferencias sustanciales. El número de profesionales de psicología y psiquiatría vinculados a las entidades promotoras de salud es inferior al de aquellos de otras especialidades; al saturarse la oferta, muchos pacientes deben esperar meses para obtener una cita o acudir a servicios particulares pagados de su bolsillo. Además, la hospitalización suele ser asumida por el Sistema General de Seguridad Social en Salud, cuya cobertura es limitada en cuanto a salud mental; tras un número restringido de días en las instituciones especializadas, el costo recae en el paciente o en su familia. En consecuencia, el acceso a la atención en salud mental continúa siendo un lujo, con un gran gasto de bolsillo que refleja desigualdades profundas ^27^.

## Gastos por enfermedad mental en Colombia

Los gastos en salud en Colombia han venido aumentando en los últimos años tanto en el sector público como en el privado. El mayor incremento en la última década se observó en los gastos de bolsillo, con un aumento cercano al 33 % entre el 2004 y el 2011 ^28^. En el caso particular de la salud mental, aunque no se dispone de cifras oficiales específicas, se estima que el gasto es considerable, dado que la mayoría de las consultas de psicología y psiquiatría son asumidas por cada usuario. Esto significa que, para acceder a la atención oportuna con profesionales capacitados, muchos colombianos deben destinar parte de su salario a las consultas privadas. Sin embargo, la capacidad de pagar no siempre garantiza el acceso, debido a la limitada disponibilidad de especialistas: en Colombia, hay apenas 4 psiquiatras por cada 100 000 habitantes, muy por debajo de la recomendación de la OMS de 10 psiquiatras por cada 100 000 personas ^26^.

Entre el 2016 y el 2019, se destinaron aproximadamente 288 millones de pesos a las estrategias de prevención, tratamiento y seguimiento en salud mental, aunque solo se ejecutó el 69 % de los recursos asignados ^29^. En el 2019, un año después de la adopción de la Política Nacional de Salud Mental, el presupuesto alcanzó su punto más alto con 115 432 millones, pero únicamente se ejecutó el 82 % ^29^. Posteriormente, en el 2022, el Ministerio de Salud destinó 8,57 mil millones para la implementación de las acciones de promoción, prevención y atención de trastornos mentales y consumo de sustancias psicoactivas ^30^. No obstante, su impacto sigue siendo incierto: en los registros se reportaron 2595 casos de suicidio en el 2021,2835 en el 2022 y 1540 en el primer semestre del 2023, con una mayor incidencia en los jóvenes ^5^. En este contexto, el acceso a la prevención y al tratamiento de la salud mental continúa viéndose como un privilegio en Colombia, limitado por la escasez de recursos económicos y de talento humano en salud.

De esta manera, se refuerza la idea de que la salud mental no depende exclusivamente de factores biológicos o clínicos, ni puede entenderse en términos dicotómicos de salud o enfermedad. Se trata de un continuo que afecta múltiples dimensiones de la vida de una persona y, por ello, exige un abordaje multisectorial y colaborativo que integre al sector laboral, educativo, de vivienda, comunitario e incluso legal, entre otros ^2^.

## Servicios de rehabilitación y empleo en personas con enfermedad mental

La evidencia científica ha demostrado que los servicios de empleo con apoyo son una estrategia efectiva de rehabilitación para las personas con enfermedades mentales graves, pues facilitan la integración comunitaria y la recuperación funcional. En Colombia, Costa Rica y Perú, se identificó la existencia de programas de inclusión laboral, pero orientados principalmente a las poblaciones con discapacidad física, visual, auditiva, cognitiva o del desarrollo, sin contemplar de manera específica a quienes sufren de enfermedades mentales ^31^.

En dicho análisis, se señalaron cinco barreras para la implementación de los servicios de rehabilitación vocacional: i) mercados laborales rígidos, ii) cabildeo insuficiente, iii) subsidios públicos que generan incentivos perversos, iv) ausencia de modelos de desinstitucionalización, y v) falta de cobertura de estos servicios por parte de los aseguradores en salud ^31^.

Además, las políticas de salud de los tres países han priorizado las intervenciones médicas sobre los componentes psicosociales, lo que ha limitado la puesta en marcha de los servicios de rehabilitación y empleo para esta población. La experiencia acumulada de los programas de inclusión laboral de personas con discapacidad no psiquiátrica constituye un punto de partida para adaptar y ensayar iniciativas de empleo con apoyo dirigidas a las personas con enfermedades mentales graves en Colombia.

La evidencia internacional sobre el modelo *Individual Placement and Support,* ampliamente implementado en los Estados Unidos y en Europa, ha demostrado que los programas de empleo con apoyo pueden duplicar las tasas de inserción laboral de las personas con esquizofrenia y trastorno bipolar, en comparación con los programas tradicionales de rehabilitación vocacional ^32^. La incorporación de experiencias, como el mencionado modelo, podrían servir de referencia para diseñar estrategias locales que reduzcan el estigma, mejoren la calidad de vida y generen evidencia sobre costo-efectividad y sostenibilidad en el contexto colombiano.

## Conclusiones

Según la OMS, la salud mental tiene un valor intrínseco en el bienestar de la persona al permitirle conectarse, funcionar, ajustarse y prosperar ^2^. Esto implica que existe una relación estrecha entre la salud mental y la productividad, tanto individual como colectivamente. Una mejor atención y cobertura en salud mental en Colombia, no solo impactaría en la calidad de vida de las personas, sino que también, contribuiría a reducir las brechas socioeconómicas mediante el aumento de la productividad, favoreciendo así la construcción de una sociedad más justa y equitativa ^33^.

El Consejo de Derechos Humanos de la Organización de las Naciones Unidas (ONU) aprobó en 2017 la resolución «Salud mental y derechos humanos», en la que se reafirmó que toda persona debe tener el más alto nivel de salud física y mental ^34^. No obstante, a pesar de este reconocimiento, la salud mental sigue siendo considerada la cenicienta de la salud pública. Las brechas socioeconómicas persistentes y crecientes generan importantes discrepancias en la calidad de la salud, tanto entre los países ricos y los pobres -con diferencias de hasta 18 años en la esperanza de vida- como dentro de los mismos países. Frente a este panorama, la OMS ha indicado que es necesario mejorar la gobernanza y la gestión de los servicios de salud para generar igualdad de importancia para la salud física y la mental.

La carga de la enfermedad mental en la región es de tal magnitud, que resulta imprescindible maximizar la eficiencia en el uso de los recursos disponibles. La forma más efectiva para lograrlo es priorizar la integración de la salud mental en la atención primaria y en la comunidad, dejando la hospitalización psiquiátrica como último recurso y no como la solución central. Esta integración requiere la capacitación de los profesionales de atención primaria para que en sus consultas realicen: tamizaje, psicoeducación, diagnóstico temprano y manejo oportuno, favoreciendo así los diferentes niveles de prevención y evitando que las personas desarrollen trastornos más graves o que su salud mental se deteriore.

Finalmente, el aumento del gasto de bolsillo en salud, y en particular en salud mental, refleja una desprotección frente al principio de universalidad del sistema de salud colombiano. Por ello, es necesario implementar programas que fortalezcan la confianza de la ciudadanía en que la cobertura pública es suficiente para el manejo de su salud mental. La atención integral en este campo no solo es fundamental para la reparación del tejido social, sino también, para avanzar hacia una sociedad más pacífica. El hecho de que el poseer una mente sana se haya convertido en un lujo, evidencia fallos en la prevención y en la promoción que, en los casos más graves, pueden derivar en desenlaces fatales como el suicidio ^5^.

## Recomendaciones en política

Teniendo en cuenta todo lo anterior, se recomienda implementar los siguientes puntos:

1. favorecer los programas de prevención y promoción en salud mental, con especial énfasis en las poblaciones de mayor vulnerabilidad, particularmente los jóvenes entre los 15 y los 29 años, en quienes la conducta suicida presenta mayor incidencia;
2. fomentar las capacitaciones en salud mental, primordialmente para los profesionales de la salud en atención primaria;
3. incluir diferentes sectores, como el educativo, en acciones que favorezcan la salud mental de la población;
4. reorientar los recursos a los programas de atención comunitaria que puedan favorecer a la mayoría de los pacientes con trastornos mentales;
5. brindar la internación psiquiátrica únicamente en aquellos casos de enfermedad aguda con gran riesgo para el paciente y su entorno, y en el menor tiempo posible;
6. favorecer una red de atención comunitaria que permita la rehabilitación de los pacientes con enfermedad mental;
7. aumentar el gasto en salud mental, reconociendo la prevalencia y los costos que las enfermedades mentales producen;
8. incluir la salud mental dentro del plan de beneficios de salud, en igualdad de oportunidades y derechos que otras situaciones de salud física;
9. ampliar las actividades de prevención, las atenciones ambulatorias, el acceso a los medicamentos y las intervenciones institucionales, y
10. favorecer el desarrollo de nuevos modelos de atención psiquiátrica institucional diferentes a los que actualmente existen en las instituciones de salud mental, ya que estos últimos perpetúan el estigma, y los procesos desestructurados y desarticulados.
